# Reverse Genetics of SARS-Related Coronavirus Using Vaccinia Virus-Based Recombination

**DOI:** 10.1371/journal.pone.0032857

**Published:** 2012-03-07

**Authors:** Sjoerd H. E. van den Worm, Klara Kristin Eriksson, Jessika C. Zevenhoven, Friedemann Weber, Roland Züst, Thomas Kuri, Ronald Dijkman, Guohui Chang, Stuart G. Siddell, Eric J. Snijder, Volker Thiel, Andrew D. Davidson

**Affiliations:** 1 Molecular Virology Laboratory, Department of Medical Microbiology, Leiden University Medical Center, Leiden, The Netherlands; 2 Institute of Immunobiology, Kantonal Hospital St. Gallen, St. Gallen, Switzerland; 3 Department of Virology, University of Freiburg, Freiburg, Germany; 4 School of Cellular and Molecular Medicine, University of Bristol, Bristol, United Kingdom; 5 Vetsuisse Faculty, University of Zürich, Zurich, Switzerland; University of Hong Kong, Hong Kong

## Abstract

Severe acute respiratory syndrome (SARS) is a zoonotic disease caused by SARS-related coronavirus (SARS-CoV) that emerged in 2002 to become a global health concern. Although the original outbreak was controlled by classical public health measures, there is a real risk that another SARS-CoV could re-emerge from its natural reservoir, either in its original form or as a more virulent or pathogenic strain; in which case, the virus would be difficult to control in the absence of any effective antiviral drugs or vaccines. Using the well-studied SARS-CoV isolate HKU-39849, we developed a vaccinia virus-based SARS-CoV reverse genetic system that is both robust and biosafe. The SARS-CoV genome was cloned in separate vaccinia virus vectors, (vSARS-CoV-5prime and vSARS-CoV-3prime) as two cDNAs that were subsequently ligated to create a genome-length SARS-CoV cDNA template for *in vitro* transcription of SARS-CoV infectious RNA transcripts. Transfection of the RNA transcripts into permissive cells led to the recovery of infectious virus (recSARS-CoV). Characterization of the plaques produced by recSARS-CoV showed that they were similar in size to the parental SARS-CoV isolate HKU-39849 but smaller than the SARS-CoV isolate Frankfurt-1. Comparative analysis of replication kinetics showed that the kinetics of recSARS-CoV replication are similar to those of SARS-CoV Frankfurt-1, although the titers of virus released into the culture supernatant are approximately 10-fold less. The reverse genetic system was finally used to generate a recSARS-CoV reporter virus expressing *Renilla* luciferase in order to facilitate the analysis of SARS-CoV gene expression in human dendritic cells (hDCs). In parallel, a *Renilla* luciferase gene was also inserted into the genome of human coronavirus 229E (HCoV-229E). Using this approach, we demonstrate that, in contrast to HCoV-229E, SARS-CoV is not able to mediate efficient heterologous gene expression in hDCs.

## Introduction

SARS is a text-book example of a novel, emerging disease that resulted from the introduction of an animal virus into the human population. The natural reservoir of the SARS virus progenitor is most likely a bat species and from here the virus was transmitted to humans, probably by a route involving a mammalian amplification host [1]. SARS was first seen in the Guangdong province of China in late 2002 and spread rapidly to a further 30 countries with more than 8000 cases reported within only a few months. The outbreak was eventually brought under control by the implementation of classical infection control measures [2].

SARS is caused by a coronavirus, a group of positive strand RNA viruses that had previously only been associated with mild upper respiratory infections in humans [3]. However, SARS-CoV infection often resulted in severe atypical pneumonia and was associated with an overall case fatality ratio of about 10% [4]. The last reported case of the SARS outbreak was in April 2004 but the threat of SARS has not disappeared. There is a real risk that another SARS-CoV could re-emerge from its natural reservoir, either in its original form or as a more virulent or pathogenic strain; in which case, the virus would be difficult to control in the absence of any effective antiviral drugs or vaccines.

Reverse genetics, the generation of mutants by recombinant DNA technology, is a powerful tool to study the biology and pathogenesis of viruses and robust systems have been developed for almost all virus families. In the case of coronaviruses, a number of alternative systems have been developed including targeted RNA recombination, the systematic *in vitro* assembly of full-length cDNA copies of coronavirus genomes and the propagation of such full-length cDNAs in bacterial artificial chromosomes [5], [6], [7], [8]. We have chosen a system that is based upon the cloning and propagation of coronavirus genomic cDNAs in vaccinia virus vectors [9]. The major advantages of this approach are that it circumvents problems associated with any instability of coronavirus cDNAs in bacterial plasmids and it allows for mutagenesis of the cDNA by a process involving homologous recombination. Initially, the mutagenesis protocol comprises two steps, essentially involving positive and negative selection of the *E. coli* guanine-phosphoribosyl-transferase (*gpt*) gene. However, as the number of vaccinia viruses with coronavirus gene-specific *gpt* inserts available in the laboratory increases, the process will be reduced to one recombination step in most cases.

It has been suggested that the initial phase of SARS-CoV infection is characterised by the lack of an adequate antiviral cytokine response, which leads to an unusually high virus load. This, combined with a background of intense chemokine upregulation, can result in the severe, age-related immunopathology seen during the period of virus clearance [10], [11]. Consistent with this idea, the SARS-CoV has been shown to encode a number of type 1 interferon antagonist proteins [12]. Also Law and colleagues have shown that human monocyte-derived dendritic cells (mdDC) upregulated both pro-inflammatory cytokines and inflammatory chemokines upon exposure to SARS-CoV but did not significantly upregulate antiviral cytokines such as type 1 interferons or interleukin 12p40 [13]. However, the SARS-CoV-dendritic cell interaction remains to be fully characterised. For example, Law *et al.* showed by electron microscopy and immunofluorescence that SARS-CoV was internalised in mdDCs and they could detect both plus and minus strand RNA in the cells, which they took as evidence of viral replication. At the same time, there was no evidence of virus production, nor did the cells exhibit cytopathic changes or signs of maturation [13]. Similarly, Spiegel et al. reported that SARS-CoV can infect mdDC cells, however virus titres declined until six days post infection suggesting that SARS-CoV replication efficiency was very low [14].

In this paper, we describe the development of a vaccinia virus-based reverse genetic system for SARS-CoV (isolate HKU-39849) and the characterization of recombinant SARS-CoV. We also describe the insertion of a reporter gene (*Renilla* luciferase) as a subgenomic transcription cassette in the SARS-CoV genome and the use of this recombinant virus to study the interaction of SARS-CoV and human dendritic cells (hDCs).

## Results

### Cloning and repair of SARS-CoV cDNA

One of the main advantages of the vaccinia-virus based reverse genetic system is that it facilitates the introduction of mutations into the coronavirus cDNA by the process of homologous recombination. However, it can also be used to repair cDNAs that contain incorrect nucleotide changes that result from RT-PCR [15] and to circumvent the cloning and propagation of coronavirus cDNAs that may be unstable or toxic in bacterial plasmids [16], [17]. In the case of SARS-CoV, we found it difficult to clone or stably propagate large cDNA sequences that contained nucleotides 11370 to 11905 in bacterial plasmids, including the low-copy plasmid pWSK29. Thus, initially, we substituted this region of the SARS-CoV cDNA with a *gpt* gene in the recombinant vaccinia virus, vSARS-CoV-5prime-gpt. Subsequently, the *gpt* gene was replaced by the appropriate SARS-CoV cDNA sequences using homologous recombination involving vaccinia virus vSARS-CoV-5prime-gpt and a plasmid DNA that contained a shorter region of SARS-CoV cDNA (nts 10808 to 12837). Homologous recombination was also used to repair a non-silent nucleotide change corresponding to SARS-CoV nt 18292 in vSARS-CoV-5prime. Sequencing analysis of the SARS-CoV insert in vaccinia virus vSARS-CoV-5prime revealed an unexpected deletion of one thymidine nucleotide, corresponding to nt 7401 of the SARS-CoV genome. Notably, this deletion was not present in the plasmid DNA that had initially been sequenced. Further analyses revealed that the deletion emerged during plasmid propagation in *E.coli*, when we prepared a large plasmid DNA stock for cloning into the vaccinia virus genome. In order to repair this deletion in the vaccinia virus vSARS-CoV-5prime, and to circumvent repeated instability of SARS-CoV sequences cloned in plasmid DNAs, we used an RT-PCR-derived fragment comprised of SARS-CoV nts 6763–7940 for homologous recombination. Similarly, homologous recombination was used to repair RT-PCR-introduced nucleotide changes in the cDNA of vSARS-CoV-3prime. These included a deletion of SARS-CoV nts 26132–26152, a point mutation at nt 26811, and a deletion of two nucleotides (27808–27809).

### SARS-CoV genome nucleotide sequence comparison

Although the cloned SARS-CoV sequence is based on SARS-CoV isolate HKU-39849, we identified a number of nucleotide differences between the published SARS-CoV HKU-39849 sequence (GenBank: AY278491) [18] and the SARS-CoV HKU-39849 RNA isolated in the Bristol laboratory (designated HKU-UOB in Table 1; GenBank: JQ316196). In total there are two silent and seven non-silent nucleotide differences (leading to six amino acid substitutions; Table 1). Interestingly, eight of these nine nucleotides in the HKU-UOB sequence match to those encoded by the SARS-CoV Frankfurt-1 isolate (GenBank: AY291315) [19]. When the HKU-UOB sequence was compared with the Frankfurt-1 sequence, eight nucleotide differences were identified, three are silent and five are non-silent. Notably, seven of these eight nucleotides in the HKU-UOB sequence match to those reported in the published SARS-CoV HKU-39849 sequence. Thus, it remains to be confirmed that SARS-CoV strains used in different laboratories actually match to the initially determined sequences. The full-length SARS-CoV cDNA cloned in the recombinant vaccinia viruses vSARS-CoV-5prime and vSARS-CoV-3prime has only four nucleotide changes compared to the HKU-39849 genomic RNA isolated in the Bristol laboratory. There are three silent nucleotide changes at positions 11304, 16325 and 16955, and one deliberately introduced silent nucleotide change (nt 20279) to create unique *SfiI* and *BglI* sites that are used for ligation of vSARS-CoV-5prime and vSARS-CoV-3prime DNA to obtain a full-length cDNA of the recombinant SARS-CoV genome (Table 1; GenBank: JN854286). Thus, the recombinant SARS-CoV cDNA had 12 nucleotide changes compared to the SARS-CoV Frankfurt-1 sequence (AY291315) reported by Thiel *et al.*, [19] and 13 nucleotide changes compared to the SARS-CoV HKU-39849 sequence (AY278491) reported by Zeng *et al.* [18].

**Table 1:** SARS-CoV genome nucleotide sequence comparison.

Nucleotide position	Nucleotide in virus isolate/recombinant virus				Codon	Gene product
	Frankfurt-1^a^	HKU-39849^a^	HKU-39849 UOB^a^	recSARS-CoV^a^		
2557	A	G	G	G	ACA (Thr) or GCA (Ala)	nsp2
2601	U	C	U	U	GUU (Val) or GUC (Val)	nsp2
3461	A	A	G	G	GAU (Asp) or GGU (Gly)	nsp3
7930	G	A	G	G	GAC (Asp) or AAC (Asn)	nsp3
8387	G	C	G	G	AGU (Ser) or ACU (Thr)	nsp3
8417	G	C	G	G	AGA (Arg) or ACA (Thr)	nsp3
11304	U	U	U	C	GAU (Asp) or GAC (Asp)	nsp6
11448	U	C	C	C	AUU (Ile) or AUC (Ile)	nsp6
13494–13495	GU	AG	GU	GU	GUU (Val) or AGU (Ser)	nsp12
16325	A	A	A	G	CCA (Pro) or CCG (Pro)	nsp13
16955	U	U	U	C	UCU (Ser) or UCC (Ser)	nsp13
18065	G	A	G	G	AAG (Lys) or AAA (Lys)	nsp14
18965	A	U	U	U	AUA (Ile) or AUU (Ile)	nsp14
19084	U	C	C	C	AUA (Ile) or ACA (Thr)	nsp14
20279^b^	A	A	A	C	GGA (Gly) or GGC (Gly)b	nsp15
24933	U	C	C	C	UUU (Phe) or CUU (Leu)	S
25569	U	A	U	U	AUG (Met) or AAG (Lys)	3a
28268	U	C	C	C	AUU (Ile) or ACU (Thr)	N
28268	U	C	C	C	UUG (Leu) or CUG (Leu)	9b

a GenBank accession number SARS-CoV Frankfurt-1: AY291315; SARS-CoV HKU-39849: AY278491; SARS-CoV HKU-39849 UOB: JQ316196 and recSARS-CoV HKU-39849: JN854286.

b Introduced nucleotide change to create *Sfi*I restriction site for cloning purposes.

### Rescue of recSARS-CoV

**Figure 1:**
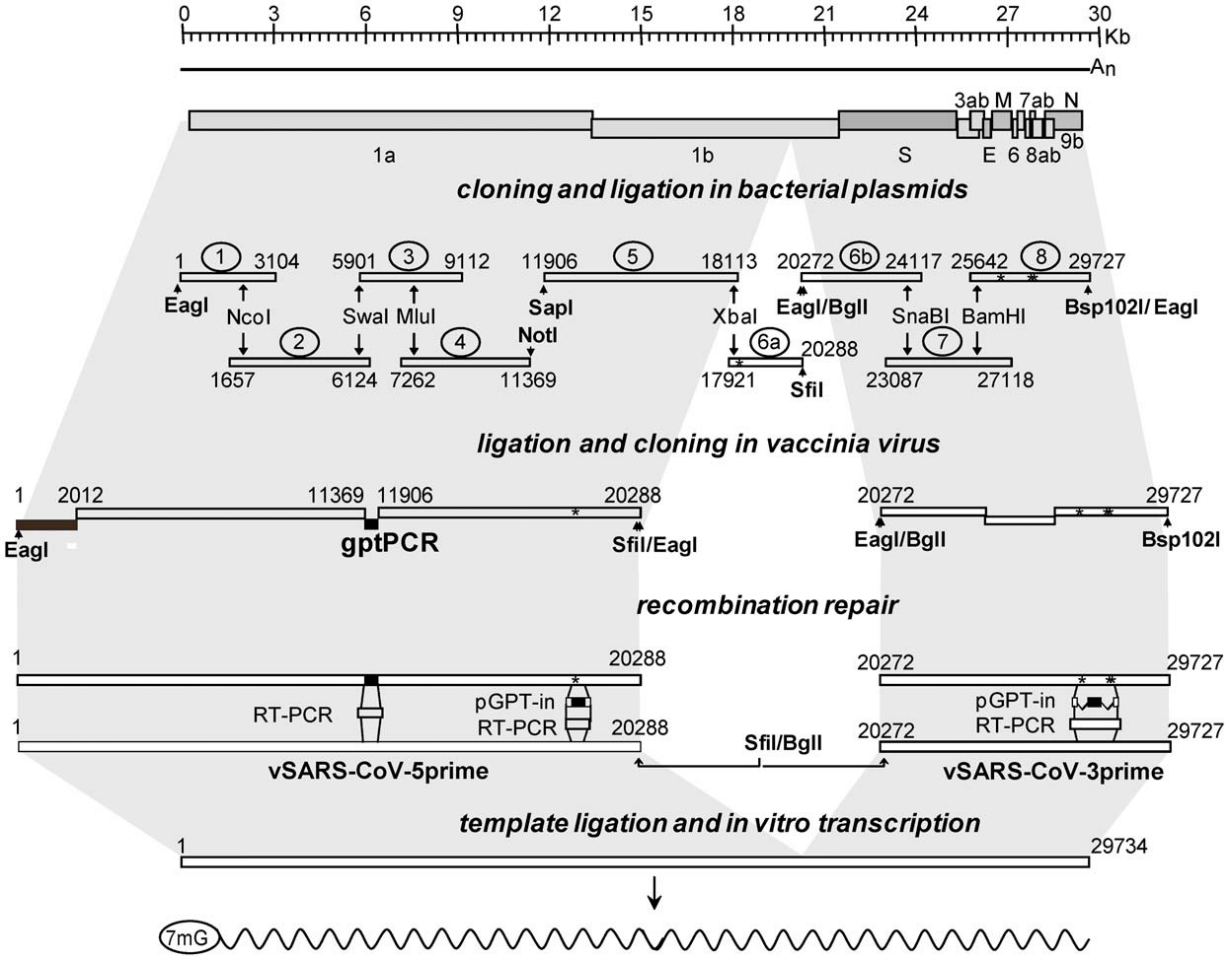
Construction of a vaccinia virus based SARS-CoV reverse genetic system. The genome structure of SARS-CoV is shown at the top of the figure. Nine cDNA clones produced from the genomic RNA of SARS-CoV isolate HKU-39849 are shown below. The region of the SARS-CoV genome encompassed by each clone is indicated by the nucleotide number (using the recSARS-CoV sequence; GenBank: JN854286) at the beginning and end of each clone. Restriction enzyme sites used to join the clones are shown, with restriction enzymes sites added to the clones shown in bold. The cDNA fragments isolated from the clones and gpt PCR products covering regions of the genome unstable as cDNA clones were ligated with each other and vaccinia virus DNA to produce two vaccinia virus recombinant clones spanning nts 1–20288 and 20272–29727 of the SARS-CoV genome respectively. The first 2012 nts of the former vaccinia virus recombinant was derived from the SARS-CoV isolate Frankfurt-1 (shaded in dark grey). Vaccinia virus mediated homologous recombination was then used to reconstitute the SARS-CoV subgenomic fragments, introducing regions of cDNA that were unstable in *E. coli* and repairing errors (*) introduced during the cloning process. This resulted in the vaccinia virus clones vSARS-CoV-5prime and vSARS-CoV-3prime. The SARS-CoV cDNA fragments were isolated from the two vaccinia virus recombinants by restriction enzyme digestion and then joined using unique *SfiI* and *BglI* sites that had been introduced into the cDNA. The ligated cDNA fragments were used as a template for *in vitro* transcription using a T7 polymerase promoter introduced at the 5′ end of the SARS-CoV 5′ cDNA clone to produce a RNA transcript representing the SARS-CoV genome.

It is now well established that the rescue of recombinant coronaviruses from infectious RNA transcripts is facilitated by the expression of the cognate N protein [7], [9], [20]. We, therefore, used a BHK-21 cell line that expressed the SARS-CoV N protein following induction with doxycycline for rescue of the recSARS-CoV [21]. Initially the cDNA inserts cloned in vSARS-CoV-5prime and vSARS-CoV-3prime were ligated to create a genome-length SARS-CoV cDNA template for *in vitro* transcription. To do this, vaccinia virus DNA derived from vSARS-CoV-5prime DNA was cleaved with *SfiI* and the vSARS-CoV-3prime DNA was cleaved with *BglI*. Both DNAs were then ligated to join the vSARS-CoV-5prime *SfiI* site with the vSARS-CoV-3prime *BglI* site (Figure 1). The resulting DNA ligation products were subsequently cleaved with *EagI*, which cuts the vSARS-CoV-3prime DNA directly downstream of the polyA sequence, and the *EagI*-cleaved DNA was then used as template for *in vitro* transcription using bacteriophage T7 RNA polymerase.

**Figure 2:**
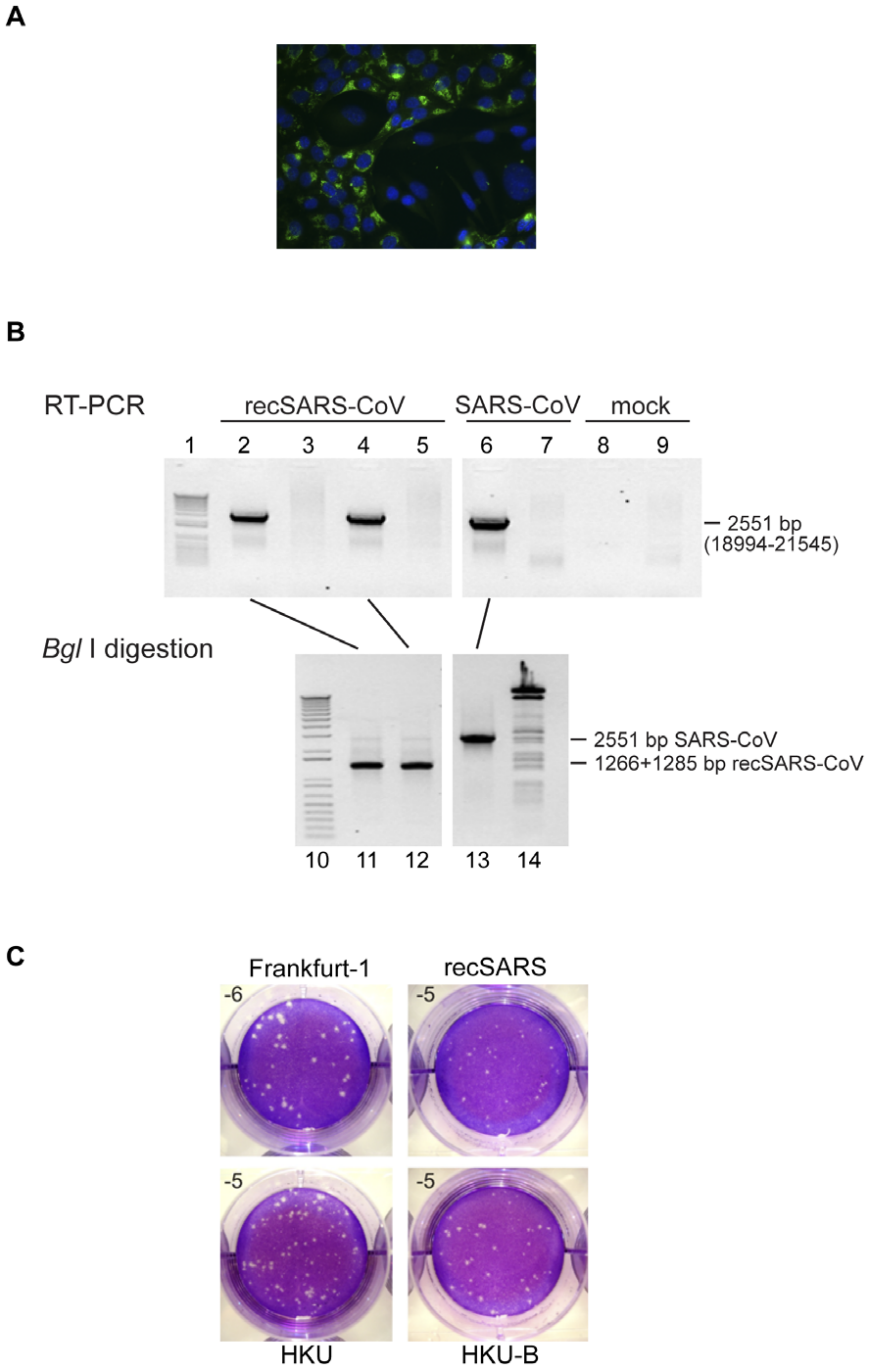
Recovery and analysis of recSARS-CoV by RT-PCR and plaque assay. **A.** Immunofluorescence microscopy analysis of Vero-E6 cells infected with P0 virus harvests obtained from cells electroporated with full-length recSARS-CoV RNA. Cells were fixed at 8 hours post infection (p.i.) and stained for nonstructural protein 3 (green) as described previously [40]. Nuclei were stained using Hoechst 33258 (blue). **B.** RT-PCR analysis and restriction enzyme digestion confirm the recovery of recSARS-CoV. Vero-E6 cells were infected with P0 culture medium from cells transfected with recSARS-CoV RNA, (harvested 48 hours post transfection, lanes 2–5), SARS-CoV Frankfurt-1 (SARS-CoV, lanes 6 and 7) or mock infected (mock, lanes 8 and 9). At 48 hours p.i., RNA was isolated from culture supernatants (lanes 4 and 5) or infected Vero-E6 cells (lanes 2, 3, 6, 7, 8 and 9). The RNA samples were used for RT-PCR analysis. Lanes 2, 4, 6 and 8 show the products obtained from RT-PCR reactions designed to amplify a genomic region containing a *BglI* restriction site that had been engineered into recSARS-CoV genome at the cDNA level, whereas lanes 3, 5, 7, and 9 show corresponding control reactions without reverse transcriptase. The RT-PCR products shown in lanes 2, 4 and 6 were then further analyzed by *Bgl*I digestion to verify the presence of this marker mutation in the recSARS-CoV progeny. The 2.5 kb PCR products derived from recSARS-CoV (lanes 11 and 12) were digested into two expected fragments of similar size (1266 bp and 1285 bp), whereas the wildtype PCR product remains undigested. The sizes of the PCR products were determined by comparison to a 1 Kb Plus DNA ladder (Invitrogen) (lanes 1 and 10). Bacteriophage λ DNA was cleaved with *Bgl*I (lane 14) as a digestion control. **C.** Comparative plaque assays of different SARS-CoV variants on Vero-E6 cells. Upon complete CPE, progeny virus was harvested from Vero-E6 cells infected with recSARS-CoV, SARS-CoV-Frankfurt-1, the original SARS-CoV HKU-39849 (HKU), and the SARS-CoV HKU-39849 used to produce the recSARS-CoV cDNAs used in this study (HKU-B). Tenfold serial dilutions were plated on Vero-E6 cells under a semisolid overlay and cell layers were fixed and stained with crystal violet after two days.

The full length SARS-CoV RNA produced *in vitro* was electroporated into BHK-SARS-N cells and the transfected cells were co-cultivated with Vero-E6 cells in a 1 to 4 ratio. After 24–48 hours, virus-induced cytopathic effects were detectable. With immunofluorescence (IF) microscopy, non-structural protein 3 and M protein could occasionally be detected in the transfected samples. However, when Vero-E6 cells were infected with passage 0 (P0) virus harvests, infection was readily detected with IF microscopy (Figure 2A). The P1 virus stock was used for subsequent experiments and had a titer of 2×10^7^ pfu/mL, approximately one log lower than that of SARS-CoV Frankfurt-1 harvested from Vero-E6 cells.

### Identification of recSARS-CoV

To ensure that the SARS-CoV that we had recovered was indeed recombinant virus, we isolated total RNA from the culture supernatant and cells that had been infected with the rescued P0 virus. We then amplified, by RT-PCR, a 2551 bp fragment that encompassed the unique *BglI* site created in the SARS-CoV cDNA by the *in vitro* ligation of the vSARS-CoV-5prime and vSARS-CoV-3prime template DNAs. Figure 2B shows that both the cell lysate and culture supernatant contained recSARS-CoV RNA that could be identified by *BglI* cleavage of the amplified RT-PCR product. As a control, we amplified the corresponding RT-PCR fragment from cells that had been infected with SARS-CoV-Frankfurt-1. The amplified fragment has the expected length but could not be cleaved by *BglI*. The RT-PCR reaction failed to produce an amplification product using RNA from mock-infected cells and PCR amplification alone failed to produce a product using the same RNA templates.

During production of the recSARS-CoV P1 stock, it was apparent that the plaque phenotype of recSARS-CoV and SARS-CoV HKU-39849 is different compared to SARS-CoV Frankfurt-1. This is illustrated in Figure 2C, which shows the plaque phenotype of recSARS-CoV, SARS-CoV Frankfurt-1, SARS-CoV HKU-39849 obtained from J.S.Peiris and SARS-CoV HKU-39849 that was recovered from the RNA used to produce the recSARS-CoV cDNAs. Clearly, the HKU-39849 lineage has a smaller plaque phenotype and replicates to lower titres compared to the SARS-CoV Frankfurt-1 virus. As shown in Table 1, there are a number of nucleotide changes between the genomes of recSARS-CoV and SARS-CoV Frankfurt-1. In addition, it is known that the SARS-CoV Frankfurt-1 virus propagated in the laboratory contains a 45 nucleotide in-frame deletion (27670–27714) in ORF7b [8], [19]. These changes may explain the different plaque sizes between the two viruses and further studies are needed to investigate this issue.

### Characterisation of recSARS-CoV in cell culture

**Figure 3:**
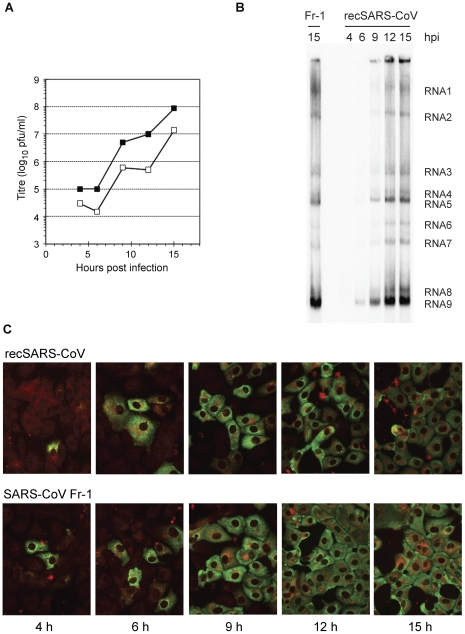
Growth curve and intracellular RNA and protein synthesis of recSARS-CoV and SARS-CoV Frankfurt-1. **A.** Vero-E6 cells were infected with a MOI of 5 for both viruses and cell culture supernatant samples harvested at the indicated time points. Virus titers were determined by plaque assay. SARS-CoV Frankfurt-1 and recSARS-CoV titers are shown as (▪) and (□) respectively. **B.** Vero-E6 cells were infected with a MOI of 5 for both viruses, and intracellular RNA was isolated at the indicated time points. Following separation in an agarose gel, hybridization with a [^32^P]-labeled DNA probe recognizing the 3′ end of all viral mRNAs was used to visualize viral RNA bands. **C.** Vero-E6 cells were infected with a MOI of 5 for both viruses and cells were formaldehyde fixed at the indicated time points. Following permeabilization, cells were double-labeled for nonstructural protein 3 (in red) and nucleocapsid protein (in green) as described previously [40].

As a next step, we characterized the replication of recSARS-CoV in cell culture by analysis of the one-step replication curve, as well as intracellular viral RNA and protein synthesis. As shown in Figure 3A, at a high MOI, the kinetics of recSARS-CoV replication are similar to those of SARS-CoV Frankfurt-1, although the titres of virus released into the culture supernatant are approximately 10-fold less. Figure 3B shows that the genome-sized RNA and 8 subgenomic RNAs are synthesized in recSARS-CoV-infected cells. Additionally, Figure 3C shows that the synthesis of viral proteins in recSARS-CoV infected cells, exemplified here by synthesis of non-structural protein 3 and the nucleocapsid protein parallels the kinetics of virus replication and is slightly delayed compared to SARS-CoV Frankfurt-1-infected cells.

### Infection of human dendritic cells

**Figure 4:**
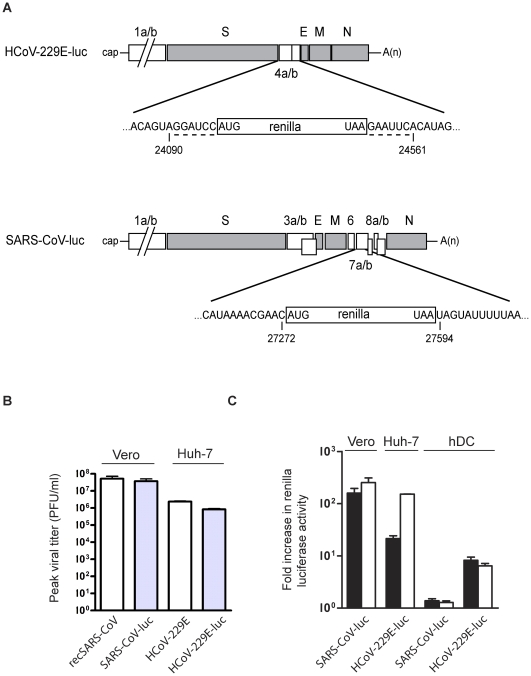
Analysis of SARS-CoV gene expression in hDCs using a recSARS-CoV expressing *Renilla* luciferase. **A.** The genome structure of recombinant SARS-CoV and HCoV-229E viruses expressing *Renilla* luciferase (HCoV-229E-luc and SARS-CoV-luc) is shown. White boxes represent ORFs encoding virus replicase and accessory proteins, grey boxes represent ORFs encoding virus structural proteins. The regions of HCoV-229E ORF4a/b and SARS-CoV ORF7a are enlarged to illustrate the ORF encoding *Renilla* luciferase and surrounding nucleotides. Nucleotide numbers depict CoV nucleotides at the border to non-CoV sequences. The dashed line in the upper panel depicts nucleotides derived from restriction sites *BamHI* and *EcoRI* that have been introduced to facilitate cloning of the recombination plasmid containing the *Renilla* luciferase gene. **B.** Pairwise comparison of the replication of recSARS-CoV and HCoV-229E with the corresponding luciferase encoding viruses. RecSARS-CoV/SARS-CoV-luc and HCoV-229E/HCoV-229E-luc were used to infect Vero-E6 and Huh-7 cells, respectively, at an MOI of 0.01. The culture supernatants were harvested at 24, 48 and 72 hrs p.i and the titers of virus in the supernatants determined by plaque assay. The peak viral titres for recSARS-CoV/SARS-CoV-luc (at 72 hours p.i) and HCoV-229E/HCoV-229E-luc (at 48 hours p.i) are shown. The average titers from 3 independent experiments are shown together with error bars. **C.** Analysis of SARS-CoV-luc- and HCoV-229E-luc-mediated *Renilla* luciferase expression in infected (MOI = 1) Vero-E6, Huh-7 and hDCs. *Renilla* luciferase expression was assessed at 12 hours (black bars) and 24 hours p.i. (white bars), The fold increase in *Renilla* luciferase expression levels in virus-infected cells represents the ratio of luciferase activity in virus-infected cells compared to that in mock-infected cells.

Infection of hDCs with SARS-CoV has been shown to be abortive and replication is only barely detectable [13], [14], [22]. However, it is not known to what extent SARS-CoV gene expression can occur in hDCs. To address this question, we used the SARS-CoV HKU-39849 reverse genetic system to construct a recombinant SARS-CoV expressing the *Renilla* luciferase by replacing the majority of SARS-CoV ORF7a (nts 27273–27594) with the *Renilla* luciferase gene (Figure 4A, termed SARS-CoV-luc). In parallel, we also constructed a recombinant human coronavirus 229E (HCoV-229E) expressing *Renilla* luciferase by replacing the majority of HCoV-229E ORF4 (nts 24091–24560; Figure 4A, termed HCoV-229E-luc). In the infected cell, production of the luciferase protein would indicate that viral genome replication has taken place and that subgenomic mRNAs have been produced and translated. Recombinant HCoV-229E was chosen to control for efficient coronavirus-mediated gene expression in hDCs because it has been shown previously that virus-like particles containing HCoV-229E-based vector RNA have the ability to transduce both mature and immature hDCs and to mediate heterologous gene expression [23]. Furthermore, efficient infection of hDCs has been demonstrated recently with recombinant HCoV-229E expressing a fusion protein comprised of the green fluorescent protein and a melanoma CD8^+^ T cell epitope (Mel-A), which efficiently activated human Mel-A-specific CD8^+^ T cells [24]. Initially, the replication of recSARS-CoV and HCoV-229E was compared to that of the corresponding luciferase expressing viruses (SARS-CoV-luc and HCoV-229E-luc) in Vero-E6 and Huh-7 cells respectively, to determine if the replacement of virus specific ORFs with the *Renilla* luciferase coding region affected virus replication in cell culture. As shown in Figure 4B, comparison of the peak viral titers for recSARS-CoV and SARS-CoV-luc and HCoV-229E and HCoV-229E-luc confirmed that the luciferase expressing viruses replicated similarly to their recombinant counterparts. SARS-CoV-luc and HCoV-229E-luc were then used to investigate the replication of SARS-CoV in hDCs. Figure 4C shows that, as expected, both SARS-CoV-luc and HCoV-229E-luc are able to mediate *Renilla* luciferase gene expression in the susceptible cell lines Vero-E6 or Huh-7, respectively. However, in hDCs only HCoV-229E-luc gave rise to significant *Renilla* luciferase expression, whereas there was no evidence that the modified recombinant SARS-CoV-luc-mediated *Renilla* luciferase expression.

## Discussion

The results presented in this paper describe the successful development of a reverse genetic system for SARS-CoV HKU-39849 that is based upon the cloning, propagation and mutagenesis of a SARS-CoV cDNA in a vaccinia virus vector. Also, we have shown that the process of vaccina virus mediated homologous recombination is not only a powerful tool to introduce mutations in the coronavirus cDNA but it is also a convenient way to repair unintentional nucleotide changes that arise during RT-PCR or plasmid DNA propagation in *E.coli*. Indeed, it is an optimal strategy to circumvent the need to clone or propagate coronavirus cDNAs in bacterial plasmids when they may be unstable or toxic. This is important because there is always the possibility that in any reverse genetic approach spurious mutations can result in the recovery of non-pathogenic viruses [25] or, conversely, they can act as compensatory mutations that allow for the recovery of viruses that would otherwise not be viable.

It is also clear from our results that the recSARS-CoV we have produced replicates to slightly lower titres compared to SARS-CoV Frankfurt-1. The SARS-CoV Frankfurt-1 isolate used in this study contains a 45 nucleotide deletion in ORF7b. It has been reported that this virus variant of the Frankfurt-1 isolate emerged during propagation in cell culture and has altered replication kinetics in specific cell lines [8], [19]. Furthermore, four nucleotide changes (A2557, U11448, U24933, U28268) have previously been identified to be specific for the Frankfurt-1 isolate [19], and these nucleotides were neither reported in the initial HKU-39849 sequence (GenBank: AY278491), nor have they been detected in the HKU-39849 RNA isolated in the Bristol laboratory (GenBank: JQ316196; Table 1). It would be worthwhile to systematically investigate the phenotypes associated with the genomic differences of recSARS-CoV and SARS-CoV Frankfurt-1, particularly, if high titres of recombinant virus are desired for *in vitro* studies. Also, in this respect, it would be important to establish the phenotype of recSARS-CoV in animal models of SARS-CoV infection [26]. The reverse genetic system for SARS-CoV-HKU-39849 will be a valuable addition to existing reverse genetic systems for SARS-CoV, because the HKU-39849 isolate has been instrumental in clarifying the aetiology of SARS [27], [28], and was used to establish important *in vivo* models for SARS-CoV infection in macaques, cats and ferrets [29], [30].

Finally, we have demonstrated that the reverse genetic system for SARS-CoV allows for the generation of recombinant viruses expressing reporter genes, such as the *Renilla* luciferase gene. The robust expression of *Renilla* luciferase following infection of Vero-E6 cells by SARS-CoV-luc provides a robust and sensitive system to study virus-host interactions and to assess putative inhibitors of SARS-CoV infection. Using this system, we show here that, in contrast to the infection of Vero-E6 cells, SARS-CoV is not able to mediate efficient heterologous gene expression in hDCs. This suggests that the abortive infection is terminated prior to the expression of detectable levels of viral gene products. This result confirms and extends the previous work of Law *et al.* and Spiegel *et al.* [13], [14], [22]. The implication is that direct infection of hDCs by SARS-CoV may only marginally contribute to the induction of any cellular immune response, in contrast to HCoV-229E, which has been shown to efficiently stimulate CD8^+^ T cells following infection of hDCs [24].

## Materials and Methods

### Virus and cells

Vero-E6 (ATCC, CRL 1586), Huh-7 (a kind gift from R. Bartenschlager, University of Heidelberg, Germany [31]) and HeLa-D980R (a kind gift from G.L. Smith, Imperial College London, UK [32]) cells were cultured at 37°C in Dulbecco's modified Eagle's medium (DMEM) supplemented with 10% fetal bovine serum (FBS), penicillin (100 units/ml) and streptomycin (100 µg/ml). Monkey kidney cells (ECACC, CV-1) and baby hamster kidney cells (ECACC, BHK-21) cells were cultured in minimal essential medium (MEM) supplemented with HEPES (25 mM), 5% FBS and antibiotics. SARS-CoV strain Frankfurt 1 was provided by H. F. Rabenau (Johann Wolfgang Goethe-University, Frankfurt am Main, Germany) and SARS-CoV strain HKU-39849 was provided by J. S. Peiris (University of Hong Kong, China). All work with infectious SARS-CoV was done inside biosafety cabinets in the biosafety level 3 facility at Leiden University Medical Center. Vaccinia virus (WR strain), vaccinia virus recombinants and fowlpox virus were propagated, titrated and purified as described previously [33].

### Isolation of PBMCs

Peripheral blood mononuclear cells (PBMCs) were derived from the blood of healthy volunteers obtained from the Bloodbank Leiden (Sanquin). PBMCs were separated from human blood by gradient density centrifugation using Ficoll (GE Healthcare). Subsequently, CD14^+^ monocytes were isolated by MACS using CD14 MicroBeads (Miltenyi Biotec). To stimulate the formation of mdDCs, the enriched cells were cultured for six days at 37°C/5% CO_2_ in RPMI 1640 medium containing 8% FCS, 2 mM L-glutamine, 800 U/ml GM-CSF (Invitrogen) and 500 U ml IL-4 (Invitrogen). Cells were analysed for the expression of iDC CD80, CD86, CD11c, CD40 and L243 (BD Biosciences) using flow cytometry.

### Plaque assays for SARS-CoV

Vero-E6 cells in 6-well clusters were infected with serial dilutions of SARS-CoV in PBS containing DEAE (0.005% w/v) and 2% FCS, and were incubated at 37°C for 1 hour. Subsequently, inocula were replaced with 2 ml of a 1.2% suspension of Avicel (RC-581; FMC Biopolymer [34]) in DMEM containing 2% FBS, 25 mM HEPES, penicillin (100 IU/ml) and streptomycin (100 IU/ml). Cells were incubated at 37°C for 48–60 hours and fixed with formaldehyde, after which plaques were visualised using crystal violet staining.

### Construction of vSARS-CoV-5prime

The genomic RNA of SARS-CoV isolates Frankfurt-1 and HKU-39849 were used to construct a set of six plasmids containing SARS-CoV-derived cDNAs encompassing nucleotides (nts) 1 to 11369 and 11906 to 20288 (Figure 1). Subsequent ligation of the cDNA clones with each other and with synthetic oligonucleotide linkers resulted in the generation of three plasmid clones. The plasmid pET5′SARS-CoV contains an *EagI* restriction site, a bacteriophage T7 RNA polymerase promoter and one additional G nucleotide upstream of the cloned SARS-CoV cDNA corresponding to nts 1 to 3104. The plasmid p234W contained SARS-CoV cDNA corresponding to nts 1657 to 11369 followed by a *NotI* site. The plasmid p56aW contained a *SapI* site upstream of SARS-CoV cDNA corresponding to nts 11906 to 20288 followed by a *SfiI* site. The larger plasmid constructs were based upon the low-copy number vector pWSK29 [35].

We initially produced a vaccinia virus recombinant, designated vSARS-CoV-5prime-gpt, that contained the *gpt* gene in place of the missing SARS-CoV cDNA (nts 11370–11905). Four cDNA fragments were prepared. Fragment 1 resulted from cleavage of pET5′SARS-CoV with *EagI* and *NcoI* (*NcoI* site at SARS-CoV nts 2012–2017), treatment with alkaline phosphatase and agarose gel purification of the 2.0 kilobase pair (kbp) fragment. Fragment 2 resulted from cleavage of p234W with *NcoI* and *NotI* and agarose gel purification of the 9.4 kbp fragment. Fragment 3 was prepared by PCR using the pGPT-1 plasmid [36] as template DNA. The PCR primers were designed to introduce *Bsp120I* and *SapI* sites at the 5′ and 3′-termini of the fragment, respectively, and following cleavage with *Bsp120I* and *SapI* and treatment with alkaline phosphatase, the 0.6 kbp fragment was purified by agarose gel electrophoresis. Fragment 4 resulted from cleavage of p56aW with *SfiI*, ligation of a linker oligonucleotide containing an *EagI* site, treatment with alkaline phosphatase, cleavage with *SapI* and agarose gel purification of the 8.4 kbp fragment. A mixture of DNA containing equimolar amounts of fragments 1 to 3 was ligated *in vitro* and the resulting product comprised of fragments 1–3 was purified following agarose gel electrophoresis. Subsequently, fragment 1–3 was ligated with fragment 4 for 2 hours and *NotI*-cleaved vNotI/tk vaccinia virus DNA [37] was added to the ligation reaction that was continued for another 16 hours at 25°C in the presence of *NotI* enzyme. The ligation products were transfected without further purification into fowlpox virus-infected CV-1 cells, and recombinant vaccinia virus was isolated as described previously [33]. Vaccinia virus-mediated homologous recombination, as described by Coley et al. [15], was used to replace the *gpt* gene that separates SARS-CoV cDNA nts 11369 to 11906 with the corresponding SARS-CoV cDNA. Finally, one RT-PCR-introduced change in the SARS-CoV cDNA (nt 18292; Figure 1) that was already present in the plasmid clone p56aW, and one single nt deletion at position 7401 that arose during plasmid DNA propagation in *E.coli*, were replaced by the wild-type SARS-CoV sequence using vaccinia virus-mediated recombination [15]. The identity of the resulting recombinant vaccinia virus vSARS-CoV-5prime was confirmed by Southern blot analysis, and, in addition, at the nucleotide level by sequence analysis.

### Construction of vSARS-CoV-3prime

Initially, the genomic RNA of SARS-CoV isolate HKU-39849 was used to construct a set of three plasmids containing SARS-CoV-derived cDNAs encompassing nts 20272 to 29727 (Figure 1). Two plasmid DNAs were subsequently fused to give rise to a cloned plasmid DNA encompassing SARS-CoV nts 20272–27118. The SARS-CoV nt 20279 of this plasmid DNA was modified to create a *BglI* restriction site (Table 1) and upstream of the *BglI* sequence an *EagI* restriction site was introduced. The plasmid DNA containing SARS-CoV nts 25642–29727 was also modified to encode a stretch of 40 adenosine nucleotides downstream of the 3′-terminal SARS-CoV nt (29727) followed by an *EagI* and a *Bsp120I* site. To insert SARS-CoV 20272–29727 into the vaccinia virus vNotI/tk genome the plasmids were first cleaved with *EagI* and *Bsp120I*, respectively, then treated with alkaline phosphatase, and finally cleaved with *BamHI* (corresponding to SARS-CoV nts 26044–26049). The resulting DNA fragments containing SARS-CoV nucleotides 20272–26046 and 26047–29727 were purified after agarose gel extraction. Subsequent ligation of the two DNA fragments at the *BamHI* ends was done for 2 hours, and *NotI*-cleaved vNotI/tk vaccinia virus DNA was added to the ligation reaction that was continued for another 16 hours at 25°C in the presence of *NotI* enzyme. The ligation products were transfected without further purification into fowlpox virus-infected CV-1 cells, and recombinant vaccinia virus was isolated as described previously [33]. Three differences in the SARS-CoV sequence, a deletion of SARS-CoV nts 26132–26152, a point mutation at position 26811, and a deletion of two nts (27808–27809), that were detected in the cloned plasmid DNA were repaired by vaccinia virus-mediated recombination [15] using a plasmid DNA containing SARS-CoV nts 25640–28808. Finally, the identity of the resulting recombinant vaccinia virus vSARS-CoV-3prime was confirmed by Southern blot analysis and, in addition, at the nucleotide level by sequence analysis.

### Construction of recombinant SARS-CoV and HCoV-229E expressing Renilla luciferase

To construct the recombinant vaccinia virus vHCoV-229E-luc, vaccinia virus vHCoV-inf-1 containing the full-length HCoV-229E cDNA [33] was recombined with the plasmid pHCoV-DCrec1 in order to replace HCoV-229E ORF4 with the *E.coli* gpt gene as previously described [24]. The resulting gpt+ vaccinia virus clone was then recombined with plasmid pHCoV-luc in order to replace the *E.coli gpt* gene with the *Renilla* luciferase gene. The identity of the resulting recombinant vaccinia virus, vHCoV-229E-luc, was verified by sequence analysis and its structure is shown in Figure 4A.

To construct the recombinant vaccinia virus vSARS-CoV-3prime-luc, vSARS-CoV-3prime was subjected to two rounds of vaccinia virus-mediated recombination. First, in order to replace SARS-CoV ORF7a, the SARS-CoV sequence 27272–27594 was replaced by the *E.coli gpt* gene using gpt-positive selection. Second, the *E.coli gpt* gene was replaced by the gene encoding *Renilla* luciferase using gpt-negative selection [15]. The identity of the resulting recombinant vaccinia virus vSARS-CoV-3prime-luc was verified by sequencing analysis and its structure is shown in Figure 4A.

### Rescue of recombinant SARS-CoV, SARS-CoV-luc, and HCoV-229E-luc

An inducible BHK-21 cell line expressing the SARS-CoV nucleocapsid (N) protein was previously constructed to facilitate the rescue of recombinant SARS-CoV [21]. To generate recombinant full-length *in vitro* RNA transcripts for electroporation into BHK-SARS-N cells, the genomic DNA of vaccinia virus vSARS-CoV-5prime was cleaved with *SfiI* and ligated for 16 hours at 25°C to *BglI*-cleaved genomic DNA of vaccinia virus vSARS-CoV-3prime or vSARS-CoV-3prime-luc. Subsequently, the ligation products were cleaved with *EagI* and used as template for *in vitro* transcription with bacteriophage T7 RNA polymerase in the presence of m7G(5′)ppp(5′)G cap analog as described previously [33]. Recombinant SARS-CoV (recSARS-CoV) or SARS-CoV-luc *in vitro* transcripts (10 µg) were electroporated into BHK-SARS-N cells as described previously [19]. The cells were then seeded out with a four-fold excess of Vero-E6 cells. After 24–48 hours, the cell culture supernatant containing recombinant SARS-CoV or SARS-CoV-luc was collected for further analysis. All experiments with live SARS-CoV were performed in the BSL-3 facility of the Leiden University Medical Center, the Netherlands.

To rescue recombinant HCoV-229E-luc, the genomic DNA of vaccinia virus vHCoV-229E-luc was cleaved with *EagI* and used as template for *in vitro* transcription with bacteriophage T7 RNA polymerase in the presence of m7G(5′)ppp(5′)G cap analog as described previously [33]. Recombinant HCoV-229E-luc *in vitro* transcripts (10 µg) were then electroporated into BHK-HCoV-N cells as described previously [19]. The cells were then seeded out with a four-fold excess of Huh7 cells. After 24–48 hours the cell culture supernatant containing recombinant HCoV-229E-luc was collected for further analysis.

### Analysis of recSARS-CoV RNA synthesis in cell culture

Vero-E6 cells were infected with SARS-CoV Frankfurt-1 or recSARS-CoV at a multiplicity of infection (MOI) of 5. Intracellular RNA was isolated as described by Van Marle *et al.* [38], separated on a 2.2 M formaldehyde-1% agarose gel, and hybridized to a 5′-[^32^P]-labeled DNA probe complementary to the 3′-terminal 794 nts of the SARS-CoV genome. RNA bands were visualized by phosphor-imaging.

### RT-PCR analysis

RecSARS-CoV was identified by the presence of a *BglI* site (nts 20272–20283) that is created by the ligation of *SfiI*-cleaved vSARS-CoV-5prime DNA and *BglI*-cleaved vSARS-CoV-3prime DNA. RecSARS-CoV RNA was isolated from Vero-E6 cells as described above and amplified by RT-PCR using the oligonucleotide primers 5′-CTCAGGCTGAAGTAGAATGG-3′ and 5′-CCGGTCAAGGTCACTACCAC-3′. The RT-PCR product (2.5 kbp) was treated with *BglI* and the reaction products analysed by agarose gel electrophoresis.

### Immunofluorescence microscopy

To monitor the progression of SARS-CoV infections, transfected cells were seeded on coverslips and fixed at various time points. Immunofluorescence assays (IFA) were done following a previously described protocol [39] using a previously described panel of SARS-CoV antisera [40]. DNA was stained with Hoechst 33258 (blue) and specimens were viewed and photographed using a fluorescence microscope (Zeiss Axioskop 2) equipped with a CCD camera.

### Luciferase assay

Cells seeded and infected in 24-well clusters were lysed in 100 µl of passive lysis buffer (Promega) supplemented with 1% Nonidet P-40. *Renilla* luciferase luminescence was measured in a LB940 Mithras ‘Research II’ (Berthold) after addition of 20 µl of substrate as described by the manufacturer.

### Nucleotide sequence accession number

The SARS-CoV HKU-39849 UOB and recSARS-CoV HKU-39849 sequences have been deposited in GenBank and have the accession numbers JQ316196 and JN854286 respectively.
